# Histological and transcriptomic analysis of adipose and muscle of dairy calves supplemented with 5-hydroxytryptophan

**DOI:** 10.1038/s41598-021-88443-w

**Published:** 2021-05-06

**Authors:** Sena L. Field, Marcela G. Marrero, Lihe Liu, Francisco Peñagaricano, Jimena Laporta

**Affiliations:** 1 Department of Animal and Dairy Sciences, University of Wisconsin-Madison, Madison, WI 53706 USA; 2 Department of Animal Sciences, University of Florida, Gainesville, Fl 32608 USA

**Keywords:** Molecular biology, Transcriptomics

## Abstract

In mammals, peripheral serotonin is involved in regulating energy balance. Herein, we characterized the transcriptomic profile and microstructure of adipose and muscle in pre-weaned calves with increased circulating serotonin. Holstein bull calves (21 ± 2 days old) were fed milk replacer supplemented with saline (CON, 8 mL/day *n* = 4) or 5-hydroxytryptophan (5-HTP, 90 mg/day, *n* = 4) for 10 consecutive days. Calves were euthanized on d10 to harvest adipose and muscle for RNA-Sequencing and histological analyses. Twenty-two genes were differentially expressed in adipose, and 33 in muscle. Notably, *Interferon gamma inducible protein-47* was highly expressed and upregulated in muscle and adipose (avg. log FC = 6.5). Enriched pathways in adipose tissue revealed serotonin’s participation in *lipid metabolism* and *PPAR signaling.* In muscle, enriched pathways were related to *histone acetyltransferase binding*, *Jak-STAT signaling*, *PI3K-Akt signaling* and *cell proliferation*. Supplementation of 5-HTP increased cell proliferation and total cell number in adipose and muscle. Adipocyte surface area was smaller and muscle fiber area was not different in the 5-HTP group. Manipulating the serotonin pathway, through oral supplementation of 5-HTP, influences signaling pathways and cellular processes in adipose and muscle related to endocrine and metabolic functions which might translate into improvements in calf growth and development.

## Introduction

Serotonin (5-hydroxytryptamine, 5-HT) is an ancient multifunctional bioamine derived from the amino acid l-tryptophan in a two-step pathway. First, l-tryptophan is converted by tryptophan hydroxylase (TPH, rate limiting enzyme) to 5-hydroxytryptophan (5-HTP), which is then converted to serotonin by an aromatic amino acid decarboxylase ubiquitous enzyme. The independent expression of two TPH enzymes enables the independent production of neuronal and non-neuronal or peripheral serotonin (TPH2 and TPH, respectively)^1^. Historically, neuronal serotonin has been thoroughly studied for its participation in behavioral functions and its relation to depression^2^, whereas more recently, peripheral serotonin is being investigated for its involvement in a plethora of biological functions^3,4^. Peripheral serotonin has been highlighted for its involvement in various physiological functions of relevance to dairy cattle biology, including regulation of milk synthesis, immune function, calcium, lipid, and energy metabolism^5–9^.

Increasing evidence supports the role of peripheral serotonin as an endocrine factor in multiple tissues to balance metabolic function^10,11^. Serotonin is synthesized and co-secreted with insulin from the pancreatic islets^12–15^. Loss of serotonin synthesis within the beta cells in mice has been shown to impair insulin secretion^16,17^. Additionally, gut-derived serotonin signals through the 5-HT2B receptor in hepatocytes to inhibit glucose uptake and stimulate liver gluconeogenesis to maintain blood glucose levels in fasted mice^18^. Increasing peripheral serotonin concentrations accelerates the metabolism of lipids, specifically through the increase of bile acid concentrations in the blood circulation of mice^19^. Adipocyte-specific *TPH1* knockout mice had decreased serotonin synthesis and reduced weight gain, suggesting a critical role for adipocyte-derived serotonin in maintaining energy homeostasis^20^. Further, in fasted mice, gut-derived peripheral serotonin stimulates white adipose tissue lipolysis and mobilizes circulating free fatty acids and glycerol by activating 5-HT2B in adipocytes^3,18^. Late-lactation non-pregnant dairy cows with increased circulating serotonin concentrations had greater circulating non-esterified fatty acid and glucose concentrations^21^.

Data primarily generated in vitro and in vivo using rodent models, suggests serotonin signaling is critical in maintaining skeletal muscle homeostasis. The skeletal muscle of rodents express the enzyme *TPH1* and various serotonin receptors indicating its ability to synthesize and respond to serotonin^22^. Feeding rats a tryptophan-free diet reduced circulating serotonin and growth hormone, exerting changes in the shape and presence of nuclei in muscle fibers, and causing lacerations to the muscle fibers, ultimately impacting body weights^23^. Additionally, in vitro studies have highlighted serotonin’s role in promoting longitudinal growth of muscle fibers^22^.

At present, there are limited studies in ruminants supporting serotonin’s metabolic role in adipose tissue, and scarce data in the muscle tissue exists. Pre-weaned dairy calves experience drastic and energy-demanding metabolic changes to allow fast growth rates. Our laboratory demonstrated that increasing circulating serotonin concentrations to growing dairy calves translated into growth improvements and increased circulating insulin concentrations^15,24^, however, no differences in glucose concentrations were found. Additionally, calves supplemented with 5-HTP had greater insulin fluorescent intensity within the islet of Langerhans in the pancreas^15^. It is important to note that 5-HTP supplemented orally can cross the blood–brain-barrier and exert metabolic effects by acting in the brain, therefore it is challenging to dissect the central vs. peripheral effects using in vivo models other than rodents. The objective of this study was to investigate cellular processes and transcriptomic alterations in adipose and muscle tissues of pre-weaned dairy calves with elevated peripheral serotonin concentrations. We hypothesize that increasing calves’ serotonin bioavailability would promote subcutaneous white adipose tissue (WAT) and gluteus medius skeletal muscle cell proliferation and growth, and trigger expression of genes related to primary endocrine and metabolic functions. Deciphering serotonin’s participation in metabolic tissues might aid in the regulation of energy-demanding metabolic changes necessary for calf growth during the pre-weaning developmental phase.

## Results

### Mapping statistics summary and data availability

RNA-Sequencing was utilized to investigate genome-wide gene expression changes in the adipose and muscle tissue of pre-weaned dairy calves after supplementation of 5-HTP, or not, for 10 consecutive days. Through Illumina sequencing roughly 27 million paired-ended reads were acquired per sample. Approximately 85% of the reads were successfully mapped to the bovine genome. Only uniquely mapped reads were considered in the analysis. The accession number GSE151781 can be used to access the sequencing data through the NCBI GEO database.

### Differentially expressed genes and pathways in adipose tissue

**Figure 1:**
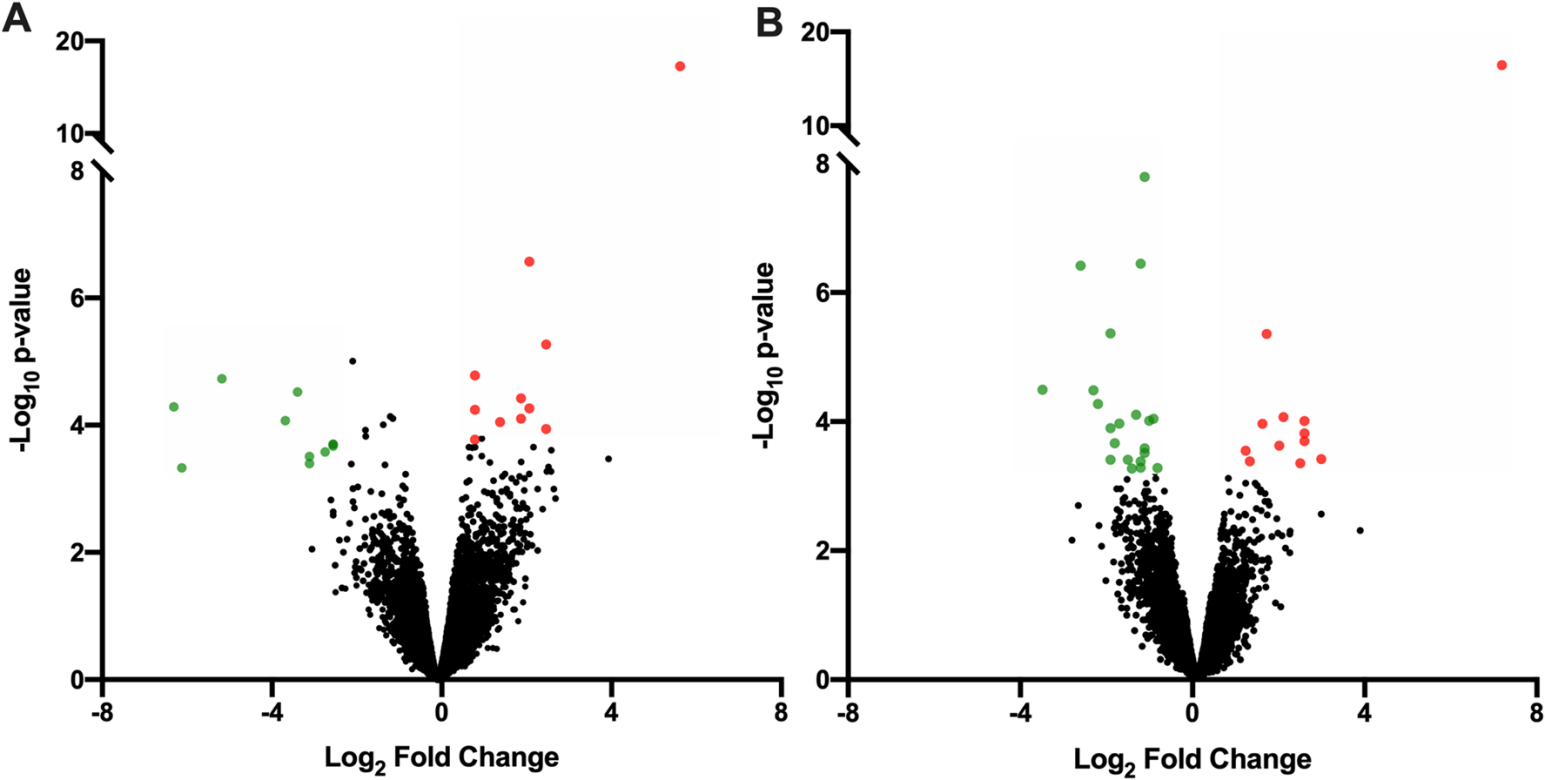
Volcano plot of differentially expressed genes in subcutaneous WAT and gluteus medius skeletal muscle tissues of pre-weaned dairy calves. Differential gene expression in the (**A**) adipose and (**B**) muscle tissues of pre-weaned dairy calves supplemented with 5-hydroxytrytophan (5-HTP, 90 mg/day; *n* = 4) or saline (CON, 8 mL saline; *n* = 4). The y-axis displays the − log10 P-value for each gene, while the x-axis displays the log_2_ fold change for that gene relative to 5-HTP. Red dots indicate upregulation and green dots indicate downregulation in the 5-HTP group (FDR ≤ 20%); black dots indicate non-significance or FDR ≥ 20%.

**Figure 2:**
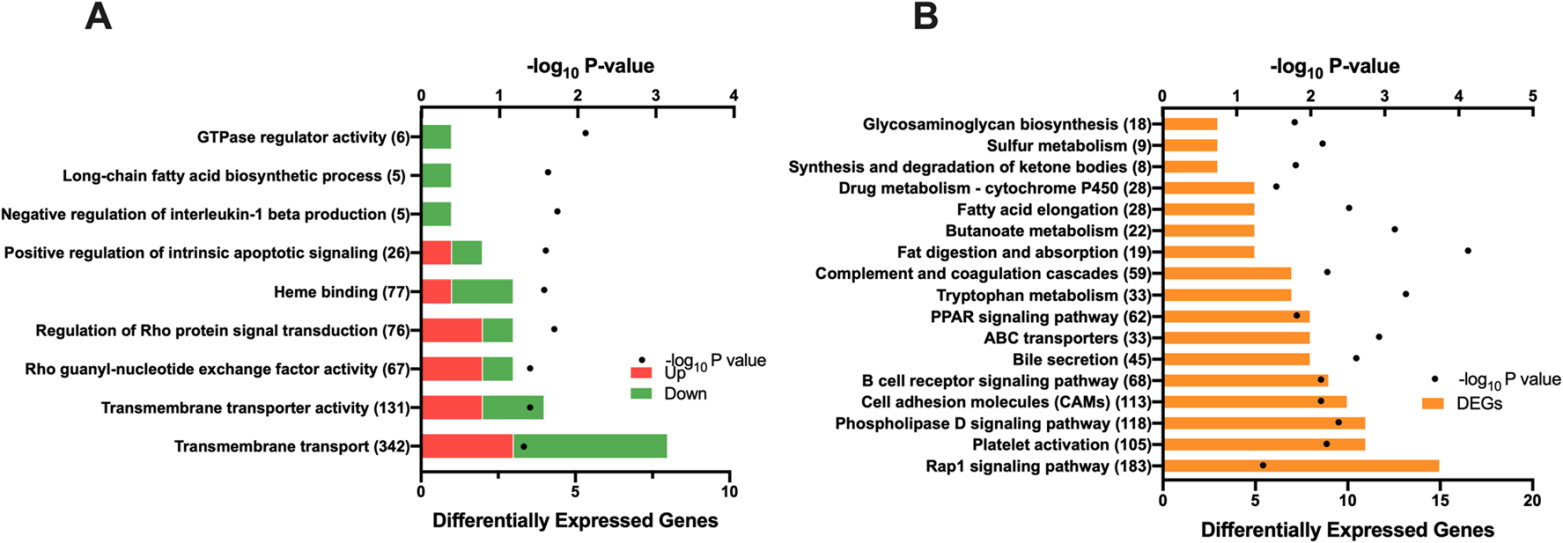
Significantly enriched (**A**) Gene Ontology (GO) and (**B**) Kyoto Encyclopedia of Genes and Genomes (KEGG)^25^ pathways in subcutaneous WAT of pre-weaned dairy calves supplemented with 5-hydroxytrytophan (5-HTP, 90 mg/day; *n* = 4) or saline (CON, 8 mL saline; *n* = 4) for 10 consecutive days. The y-axis displays the names and total number of genes in each pathway/term, and the x-axis displays the number of differentially expressed genes (DEGs) within each pathway/term and the significance of enrichment (− log10 P-value). Red and green bars display the number of upregulated and downregulated DEGs in the 5-HTP group; orange bars display the number of DEGs.

Twenty-two genes were differentially expressed (DEGs) between groups, of which 11 were upregulated and 11 were downregulated in 5-HTP-supplemented calves relative to non-supplemented calves (False Discovery Rate (FDR) ≥ 20%, Fig. 1A; Supplemental Table 1). The greatest upregulated DEG in adipose was *IFI47* (*Interferon gamma inducible protein-47*, log FC = 6.2; *P* = 5.21 × 10^–18^). Nine Gene Ontology (GO) pathways were identified (Fig. 2A). The GO pathways with the highest number of DEGs was *transmembrane transport and activity,* followed by *regulation of Rho protein signal transduction* and *Rho guanyl-nucleotide exchange factor activity*. Pathways with only downregulated genes include *GTPase regulator activity*, *long-chain fatty acid biosynthesis* and *negative regulation of interleukin-1 beta production*.

Seventeen Kyoto Encyclopedia of Genes and Genomes (KEGG) pathways were identified in adipose tissue (Fig. 2B). KEGG pathways with ten or more DEGs include *cell adhesion molecules, phospholipase D signaling pathway, platelet activation* and *Rap1 signaling pathway.* Other enriched pathways of interest with less than ten DEGs include *synthesis and degradation of ketone bodies, tryptophan metabolism, B-cell receptor* and *PPAR signaling pathway.*

### Differentially expressed genes and pathways in muscle tissue

**Figure 3:**
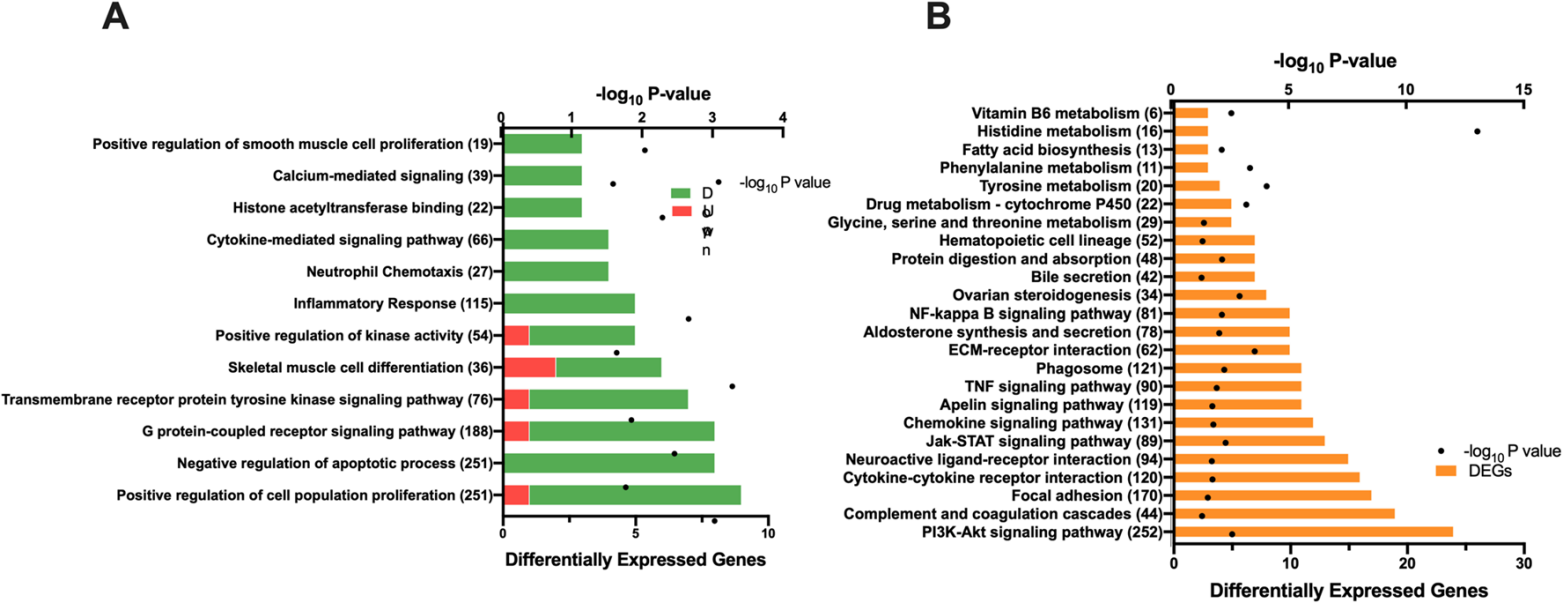
Significantly enriched (**A**) Gene Ontology (GO) and (**B**) Kyoto Encyclopedia of Genes and Genomes (KEGG)^25^ pathways in the gluteus medius skeletal muscle tissue of pre-weaned dairy calves supplemented with 5-hydroxytrytophan (5-HTP, 90 mg/day; *n* = 4) or saline (CON, 8 mL saline; *n* = 4) for 10 consecutive days. The y-axis displays the names and total number of genes in each pathway/term, and the x-axis displays the number of differentially expressed genes (DEGs) within each pathway/term and the significance of enrichment (− log10 P-value). Red and green bars display the number of upregulated and downregulated DEGs in the 5-HTP group; orange bars display the number of DEGs.

Thirty-two genes were DEGs, of which 11 were upregulated and 21 were downregulated in 5-HTP-supplemented relative to non-supplemented calves (FDR ≥ 20%, Fig. 1B, Supplemental Table 2). The greatest upregulated DEG in muscle was *IFI47* (*P* = 2.49 × 10^–19^). Twelve GO pathways were identified (Fig. 3A). Interestingly, the majority of GO pathways contained DEGs that were downregulated by 5-HTP. Pathways with all downregulated DEGs include immune related pathways (i.e., *cytokine-mediated signaling*, *neutrophil chemotaxis* and *inflammatory response*), pathways related to cell cycle (i.e., *positive regulation of smooth muscle cell proliferation* and *negative regulation of apoptotic process*) and histone modifications (i.e., *histone acetyltransferase*). Specific downregulated genes of the *Histone acetyltransferase binding* pathway, including *EGR1*, *NR4A3* and *TRIM68*. GO pathways and terms such as *skeletal muscle cell differentiation*, *G protein-coupled receptor signaling positive regulation of kinase activity*, *transmembrane receptor protein tyrosine kinase signaling*, and *positive regulation of cell population proliferation* contained a higher percentage of downregulated DEGs, with 1 or 2 upregulated genes*.*

Twenty-four KEGG pathways were identified in the muscle tissue (Fig. 3B). Pathways with ten or more DEGs include *PI3K-Akt signaling pathway, Jak-STAT signaling pathway, TNF signaling pathway* and *NF-kappa B signaling pathway.* Other pathways of interest include *hematopoietic cell lineage, fatty acid biosynthesis* and *amino acid metabolism,* including *histidine, tyrosine, glycine, serine, and threonine.*

### Validation of RNA-Seq results with quantitative RT-PCR

Five DEGs in adipose (*ANKRD33B, CYP4F, IFI47,* and *REEP6*) and four DEGs in muscle (*CISH, CCN2, EGR1*and *FOS*) were selected for validation. The assessment of expression by qPCR for all nine genes followed the same direction of expression and similar − log_2_ fold change as that obtain by RNA-Seq (Supplementary Fig. 3). Refer to Supplementary Table 3 for primer sequences and Supplementary methods for qPCR methodology.

### Histological evaluation of tissue microstructure

**Figure 4:**
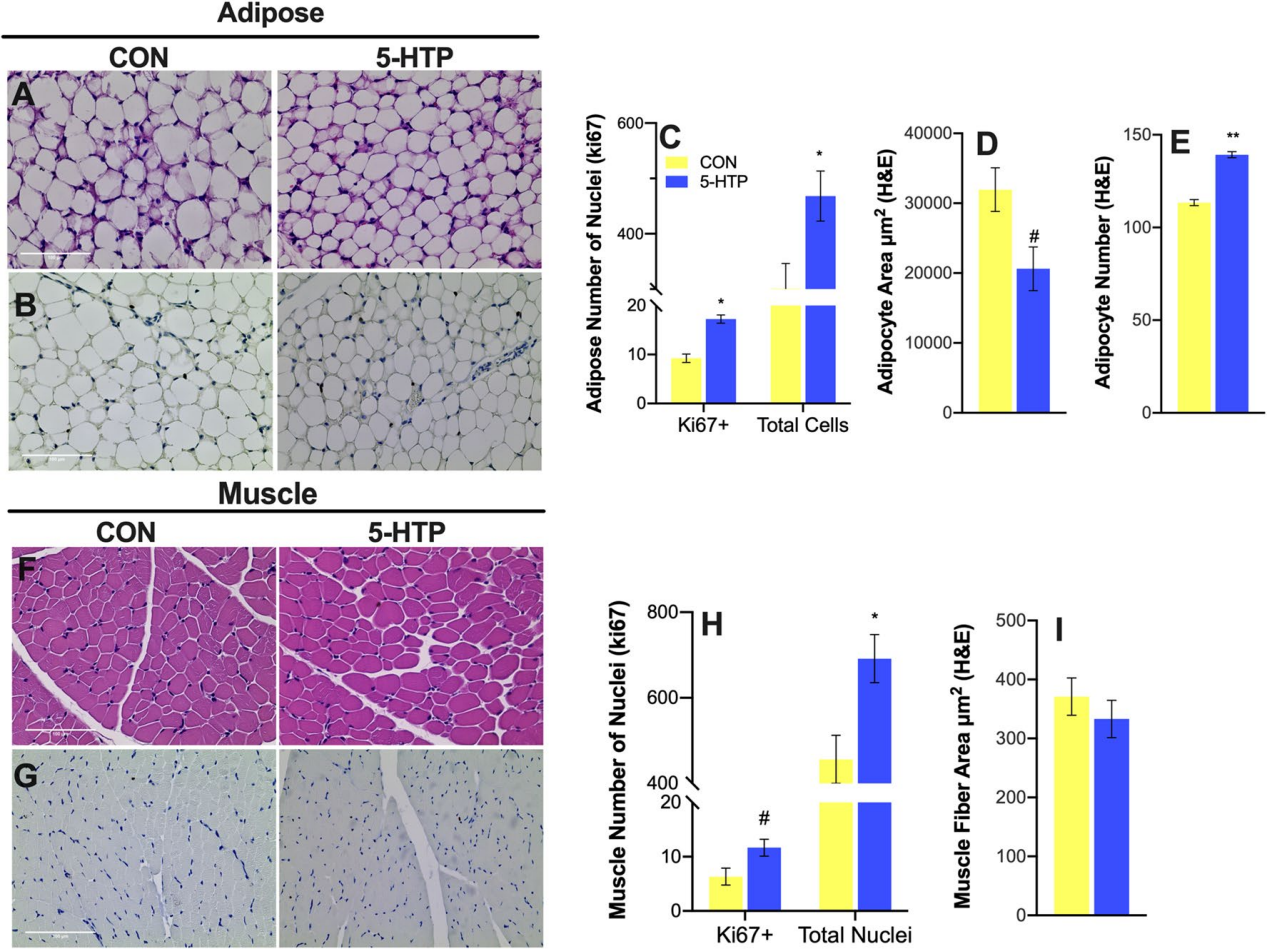
Histological evaluation of subcutaneous WAT and gluteus medius skeletal muscle tissues of pre-weaned dairy calves supplemented 10 days with 5-Hydroxytrytophan (5-HTP, 90 mg/day; *n* = 4) or saline (CON, 8 mL saline; *n* = 4). (**A**) Hematoxylin and eosin staining of adipose tissue and (**B)** representative immunohistochemical ki67 staining of adipose tissue to quantify (**C**) adipose ki67-positive nuclei and total nuclei, (**D**) adipocyte cross sectional area and (**E**) adipocyte number. (**F**) Hematoxylin and eosin staining of muscle tissue and (**G**) representative immunohistochemical ki67 staining of muscle tissue to quantify (**H**) ki67-positive nuclei and total nuclei and (**I**) muscle fiber cross sectional area. Total tissue area for photomicrographs taken at ×40 is 96,820 µm^2^. Asterisk (*) denotes P ≤ 0.05 and pound sign (#) indicate tendencies 0.05 < P ≤ 0.10 between CON vs 5-HTP treatment groups.

Hematoxylin and eosin staining of adipose and muscle tissue was preformed to visualize the tissue microstructure (Fig. 4A,F, respectively) and quantify adipocyte number, adipocyte and muscle fiber cross sectional area. Adipocyte cross sectional area tended to be reduced (*P* = 0.08, Fig. 4D), resulting in more adipocytes per field, in 5-HTP-supplemented relative to CON calves (*P* = 0.001, Fig. 4E). Cross sectional area of muscle fibers was not impacted by 5-HTP supplementation (*P* = 0.4, Fig. 4I).

The ki67 assay was conducted to visualize and quantify the number of proliferating cells (ki67-positive, stained brown) and distinguish these from non-proliferating cells (ki67-negative, stained blue) in adipose and muscle tissue (Fig. 4B,G, respectively). In adipose tissue, the number of positive ki-67 nuclei and the total number of nuclei were greater in 5-HTP-supplemented relative to CON calves (*P* < 0.03; Fig. 4C), although the proportion ki67-positive/total nuclei was not different between treatments (*P* = 0.21, data not shown). In the muscle tissue, 5-HTP-supplemented calves tended to have greater ki67-positive nuclei and a greater number of total nuclei per field relative to CON (*P* = 0.07 and *P* = 0.04, respectively, Fig. 4H), although the proportion ki67-positive/total nuclei was not different between treatments (*P* = 0.5, data not shown).

## Discussion

The pre-weaning period of dairy calves is characterized by a dramatic shift in energy as the animal grows and evolves from a functional monogastric to a ruminant^26^. This energy demanding developmental window is crucial for optimal calf growth, development and future performance^27^. Peripheral serotonin is well established in the human and murine model for its involvement in regulating energy metabolism^16,28–30^, however, the role of this biogenic amine in dairy cattle is just beginning to be elucidated^15,31^. Exploring serotonin’s role in the regulation of cellular and molecular processes in metabolic tissues during the pre-weaning period prompted the current study. Increasing peripheral serotonin in pre-weaned dairy calves is attainable through the oral supplementation of 5-HTP (Supplementary Fig. 2)^24^. This is the first in vivo evidence of serotonin’s contribution to energy-regulating mechanisms in the adipose and skeletal muscle of growing dairy calves.

The adipose tissue is a vastly complex endocrine, immune and highly active metabolic organ with the capacity to store energy, specifically through the increase in cell number (hyperplasia) and adipose cell size (hypertrophy)^32^. Adipocytes stay in a dynamic state depending on energy intake and expenditure^33^. In windows of chronic positive energy balance, adipocytes expand in size as they store surplus energy in the form of triglycerides^33^. The role of peripheral hormones in the regulation of lipid metabolism is well known. Insulin antilipolytic effect, through the downregulation of cyclic adenosine monophosphate, reduces the release of fatty acids from mature adipocytes promoting its expansion^34^. On the other hand, hormones such as epinephrine and glucagon bind to specific receptors on the adipocyte to promote fatty acid mobilization, thus reducing adipocyte size^35,36^. Literature highlighting peripheral serotonin’s role in the regulation of lipid metabolism is emerging, but vary greatly between species. Fasted mice subjected to intraperitoneal serotonin injections, had decreased plasma triglyceride and non-esterified fatty acid (NEFA) concentrations^19^. In fasted sheep, intravenous injections of serotonin increased circulating triglyceride and NEFA concentrations^37^. In late-lactation cows, intravenous infusion of 5-HTP increased NEFA concentrations in a dose dependent manner^21^.

The enrichment of multiple pathways related to lipid metabolism (e.g., *long-chain fatty acid biosynthesis, fatty acid elongation, fat digestion and absorption and synthesis and degradation of ketone bodies*) in our experiment, support the involvement of serotonin in lipid metabolism in the bovine. *Fatty acid elongation* consisted of five DEGs (*ELOV1, ELOV3, MECR, HADHA* and *ACOT2*), which code for enzymes involved in fatty acid elongation cycle as well as the mitochondrial beta-oxidation pathway, the major-energy producing process in tissues breaking down fatty acids into acetyl-CoA^38^. The beta-oxidation pathway is characterized by breaking down long chain fatty acids that have been converted to acetyl-CoA into smaller acetyl-CoA chains to enter the citric acid cycle. Recently discovered in roundworms, blocking the entry of fatty acyl coenzyme A into peroxisomal beta-oxidation in the periphery impacted neuronal serotonin and ultimately disrupted metabolic signals of satiety^39^. Central serotonin is well-known for its role in the control of satiety by interacting with orexigenic and anorectic peptides^40^, however, complex interactions of peripheral serotonin acting directly or indirectly in the control of satiety are developing. A 10-day supplementation of 5-HTP increased calves’ average daily gain (Supplementary Fig. 4)^24^. It is important to mention that orally supplemented 5-HTP might have crossed the blood brain barrier directly impacting neuronal synthesis of serotonin. Research investigating the mechanisms by which peripheral serotonin bioavailability might independently regulate satiety are needed and could have a significant impact on the improvement of feed efficiency in dairy cattle.

Adipogenesis is a tightly regulated cellular differentiation process where hyperplasia is mainly regulated through transcription factors, such as PPAR-γ and CCAAT enhancer-binding proteins (C/EBPs). Adipose-derived serotonin autocrine/paracrine actions are necessary for adipocyte differentiation, specifically through the 5-HT2A and 2B receptors^41^. Antagonists of the 5-HT2A and 2B receptors inhibit adipogenesis in preadipocyte cells to mature adipocytes. The TPH1 protein is also expressed in adipocytes and is required for cell differentiation^41^. In humans, serotonin metabolites activate and bind peroxisome proliferator-activated receptor γ (PPAR-γ) to regulate the differentiation from preadipocyte to adipocyte^42^. In vitro, *TPH1* knock down in 3T3-L1 pre-adipocyte cells decreased expression in PPAR-γ, which directly impaired adipogenesis^41^. In our study, increasing peripheral serotonin concentrations enriched the *PPAR signaling pathway* with differential expression of two key genes, namely perilipin 4 (*PLIN4*) and apolipoprotein A1 (*APOA1*). PLIN4 is a member of a family of proteins notable for protecting lipid droplets in adipocytes and is essential for the mobilization of fatty acids form adipose tissue. APOA1 is a structural protein that constitutes high-density lipoprotein which assists in transporting cholesterol and phospholipids from tissues to the liver to be excreted^43^. This, along with the reduction in adipocyte surface area, might indicate that serotonin triggers mobilization of free fatty acids from mature adipocytes. However, circulating NEFA concentrations were not elevated in 5-HTP-supplemented calves (Supplementary Fig. 2), which might indicate that free fatty acids are being metabolized by other peripheral tissues.

The modulation of energy-related pathways by serotonin could be of importance to improve energy metabolism during the metabolically stressful pre-weaning period for growing calves. In the adipose tissue, an upregulation of the pathway *regulation of Rho protein signal transduction* and an enrichment of the pathway *Rap1 signaling* and *cell adhesion molecules (CAMs)* occurred after serotonin concentration was increased for 10 days. Ras homologous (Rho, Ras, Rab) protein superfamily is a member of the Ras small GTPases that act as “molecular switches” and are key regulators of many cellular events such as cell proliferation, differentiation, migration or apoptosis. In a process termed serotonylation, serotonin covalently couples by transglutaminases to two small GTPases, Rab3a and Rab27a, to regulate insulin secretion in the pancreas^13^. Rap1 are small GTPase cytosolic proteins that regulate cellular functions such as cell polarity, cell–cell junction formation, and cell adhesion. It is plausible that serotonin is being taken up by adipocytes, acting upon small GTPases, and regulating molecular changes such as adipocyte proliferation and size. Although this speculation is advanced, we cannot rule out the possible intracellular actions of serotonin in adipocytes.

Increasing peripheral serotonin concentrations also modulated cytosolic proteins that are vital for effective intracellular signal transduction. Out of the six genes in the *GTPase regulator activity* pathway in the adipose tissue, one DEG (*GPSM1*) was downregulated. Interestingly, *GPSM1* (G- protein signaling modulator 1), also known as AGS3 (activator of G protein signaling 3), is recognized to play diverse and functional roles in cell division and lipid metabolism by regulating the phosphorylation of cyclic AMP response element-binding protein^44,45^. Activator of G protein signaling 3 (AGS3) null mice have reduced whole-body fat content^44^. It is possible that serotonin regulation of cyclic AMP or other cytosolic proteins, might influence intracellular signaling cascades resulting in the modulation of metabolic related processes in the adipose tissue.

Literature investigating serotonin’s role in skeletal muscle growth and metabolism is evolving. The skeletal muscle is a highly specialized metabolic tissue regulated by endocrine and autocrine signals, like growth hormone (GH). Growth hormone is necessary for postnatal growth through the stimulation of protein synthesis in cattle^46^. Serotonin has been shown to increase growth hormone secretion from the posterior pituitary^47^. Although literature directly linking the role of skeletal muscle-derived serotonin in muscle metabolism and growth is lacking, it is possible that these hormones synergistically modulate skeletal muscle metabolism and homeostasis. It is also possible that 5-HTP direct actions in the brain might have influenced the synthesis and secretion of hormones in the pituitary gland. Serotonin is known for its involvement in the regulation of vascular smooth muscle and possesses both vasoconstrictor and vasodilator properties^48^. In the gastrointestinal tract, serotonin mediates the contraction and relaxation of smooth muscle^49,50^. In leeches, serotonin has been shown to enhance striated muscle mechanical performance^48^. More recently, in vitro myostatin knockout mouse gastrocnemius muscle showed that serotonin concentrations and the *TPH1* gene are present in the skeletal muscle and act to promote muscle growth through the increase in longitudinal growth of muscle fibers^23^. In the present study, increasing serotonin concentrations modulated muscle-derived genes related to *positive regulation of smooth muscle cell proliferation, positive regulation of cell population proliferation* and *skeletal muscle cell differentiation.* This might indicate serotonin plays a role in regulating cell proliferation in the bovine skeletal muscle, although it is not possible to discern whether this observation is due to a direct serotonin action in the muscle tissue or indirect through modulation of the GH-IGF-1 axis.

Serotonin has been shown to promote proliferation of bovine pulmonary artery smooth muscle cells in vitro^51^. These authors propose that serotonin acts both intracellularly and through cell surface receptors in combination with an observed increase cyclic AMP activation. Herein, we observe increased cell proliferation in the muscle of calves with increased serotonin concentrations. Within the *skeletal muscle cell differentiation* pathway, the *FOX11* and *MYF6* genes were upregulated. Myogenic factor 6 (MYF6) is part of a family of transcription factors that are known for their involvement in muscle differentiation, regeneration and satellite cell specification^52^. The upregulation of *MYF6* and the increase in skeletal muscle cell proliferation may translate into the improved growth rates observed for the calves in our study^24^. However, we cannot determine whether the increased cell proliferation of skeletal muscle is a direct action of intracellular serotonin or through the activation of cell surface receptors and downstream signaling cascades inducing proliferative genes.

There are seven serotonin receptor families with more than twelve subtypes, which primarily consist of G-protein coupled receptors (GPCR). Depending on which receptor subtype is activated upon ligand binding, intracellular alpha proteins, G_s_, G_q/11_, or G_i/o_, elicit different signaling cascades to modulate the activity of proteins and gene transcription^53^. The enrichment analysis in the muscle tissue revealed significant pathways such as *TNF*, *Jak-STAT*, and *PI3K-AKT* signaling. Serotonin receptors employ numerous transduction mechanisms per each different receptor subtype. Enrichment of downstream signaling molecules such as the *PI3K-AKT* pathway are involved in the regulation of cell proliferation and stimulation of growth. Even though differences in serotonin receptor expression were not captured in the muscle of these calves (Supplementary Fig. 1), the enrichment of the PI3K-AKT pathway which is activated downstream of serotonin binding to all its receptors, might be indicative of increase activity of the receptor.

Novel research has uncovered epigenetic roles of serotonin. Serotonin can translocate to the nucleus and modify histone 3, which enhances TFIID (transcription factor II D) binding and modulate gene expression^54^. This novel discovery has uncovered vast roles of intracellular serotonin independent of receptor binding. The *histone acetyltransferase binding* pathway was enriched in the muscle tissue consisting of three downregulated genes (*EGR1*, *NR4A3*, *TRIM68*). EGR1 is a transcriptional regulator with major roles in cell survival, proliferation and cell death. Histone acetylation results in a more relaxed chromatin structure, which allows for greater levels of gene transcription^55^. The downregulation in *histone acetyltransferase binding* genes observed in our study might lead to transcription repression. Interestingly, the majority of the genes in the muscle tissue were downregulated by 5-HTP supplementation. It is possible that serotonin intracellular actions in the nucleus might modulate gene expression in the muscle^54^.

Peripheral serotonin can act as an immunomodulator^8,56,57^ and can be synthetized by leukocytes and immune organs, such as the spleen and thymus in dairy calves^58^. We have shown that bovine peripheral leukocytes express serotonin receptors and genes coding for the serotonergic machinery responsible for serotonin synthesis, uptake and metabolization^58^. Indeed, increasing peripheral serotonin in the neonate bovine led to an upregulation of serotonin receptors and cytokines in peripheral leukocytes. Skeletal muscle cells are also an important source of cytokines, which can regulate energy metabolism and modulate immune function^59^. Specifically, cytokine signaling from myoblasts play a prominent role in regeneration and hypertrophy^60^. In the present study, increasing serotonin concentrations downregulated genes in immune-related pathways, such as *cytokine-mediated signaling pathway, neutrophil chemotaxis,* and *inflammatory response*. It is possible that serotonin is acting directly in the muscle to downregulate the expression of genes related to immune signaling. These findings are in conjunction with the human and murine model and suggest that serotonin might play an immunoregulatory role in the neonate bovine. The exact mechanism by which peripheral serotonin modulates skeletal muscle immune genes is unclear. Future elucidation of the molecular mechanisms behind serotonin immunomodulatory effects and its role in orchestrating innate and adaptive immunity might contribute to the overall health status of growing animals.

Interferon-γ (IFN-γ) is a pro-inflammatory cytokine which triggers the JAK/STAT intracellular signaling pathway to regulate the expression of more than 1000 genes^61^. Interferon-γ inducible protein 47 (*IFI47*) was differentially and highly expressed in both the adipose and muscle tissue of 5-HTP supplemented calves. *IFI47* encodes for an intracellular GTPase protein family, known as p47 GTPases, which are bound to lipid membranes and are responsible for regulating immunity in resistance to intracellular pathogens^62^. The p47 GTPase family is a group of six proteins that are produced in response to interferons^62^. p47 GTPase proteins act as an immune defense mechanism to intracellular infection. Although serotonin directly promotes IFN-γ production in the presence of monocytes in humans^63^, the interplay between IFN-γ, serotonin and p47 GTPase proteins in the bovine is unknown. Thus, it is possible that serotonin might be increasing IFN-γ and indirectly inducing *IFI47* expression aiding dairy calves’ immune competence and their adaptive immune system development. This is of relevance due to calves being born with a naïve immune system that develops continuously with age^64^. The association between serotonin and p47 GTPase proteins could open new avenues of research exploring this biogenic amine in bovine innate and adaptive immune defense.

There are several limitations to our study including the small sample size and the inability to discern central from peripheral effects of orally supplemented 5-HTP. In addition, we cannot determine whether the observed changes result from a direct action of serotonin on the adipose and muscle, or indirectly through its actions in other metabolic tissues such as the liver and pancreas. The increased circulating insulin concentrations observed in these calves might be impacting the adipose and muscle transcriptome and cellular processes indirectly. Future work will focus on dissecting the direct contributions of serotonin actions in metabolic tissues such as the adipose, muscle, liver, and pancreas.

## Conclusions

Increased peripheral serotonin signaling induced microstructural changes in the adipose tissue reducing adipocyte surface area and increasing proliferative and total cell number. While muscle fiber surface area was not impacted, greater proliferative nuclei and a greater number of total nuclei was observed. Our data highlights tissue specific genes and pathways impacted by increased peripheral serotonin. In the adipose, serotonin impacted the expression of genes involved in pathways related to intracellular signal transduction mechanisms, lipid metabolism and fatty acid biosynthesis, cell proliferation and differentiation. In muscle, serotonin primarily downregulated genes involved in immune signaling, cell cycle regulation, and histone modification. IFI47 revealed as a candidate gene for future exploration, as a potential serotonin inducible gene with an immune role involved in metabolic crosstalk. Future studies will elucidate the mechanistic links of peripheral serotonin in metabolic tissues which might confer improved metabolism and growth to the dairy calf.

## Methods

This experiment was conducted under protocols approved by the Institutional Animal Care and Use Committee at the University of Florida. All methods were performed in accordance with the relevant guidelines and regulations, and the study is reported in accordance with ARRIVE guidelines. Holstein bull calves (*n* = 8, 18 ± 2 day old, 47 ± 3.2 kg BW) were housed in a shaded barn in straw bedded pens. Calves received 4 L of milk replacer (Southeast Milk Inc, Okeechobee, FL) at 7:00 a.m. and 7:00 p.m. during a 5-day adaptation period. Water and grain (Purina Animal Nutrition LLC, Shoreview, MN) were offered ad libitum for the 15 days. After the adaptation period, treatments were administered for 10 consecutive days by supplementing the 7:00 a.m. milk replacer with 5-HTP (90 mg, 1.8 mg/kg, *n* = 4, Sigma, St. Louis, MO; #H9772) or saline (CON, *n* = 4). The chemical composition of the milk replacer and concentrate, and serotonin concentrations can be found in Marrero et al.^24^.

The morning after the 10th day of supplementation, calves were euthanized to harvest major organs and tissues, including muscle and adipose. Calves received an intravenous injection of 0.2 mg/kg of xylazine followed by captive bolt and jugular exsanguination. Subcutaneous white adipose tissue (WAT) near the tail head and pelvis, and skeletal muscle tissue from gluteus medius (rump) were harvested. Approximately 1 cm^3^ were washed in sterile PBS and stored in RNA*later* (ThermoFisher, Invitrogen; #AM7020, Grand Island, NY) overnight at 4 °C and at − 80 °C until RNA isolation. For histological analyses tissue samples (~ 0.5 cm^2^) were placed in 4% paraformaldehyde at 4 °C overnight. Tissue was rinsed in increasing concentrations of ethanol (25%, 50%, 70%, and 100%), and then transferred to 70% ethanol and stored at 4 °C until embedded in paraffin and sectioned using a microtome (MICROM GmbH, MH325, Walldorf, Germany) at 5 μm onto poly-l-lysine coated slides.

RNA-Seq library was constructed using the NEBNext Ultra II RNA Library Prep Kit for Illumina (#E7775; New England BioLabs, Ipswich, MA) following the manufacturer’s protocol. The libraries were sequenced using the Illumina NovaSeq 6000 platform (Illumina Inc., CA) which generated 150 base-pair paired-end reads. Sequencing reads were tested for quality before and after trimming using the software FastQC (version 0.11.7, Babraham Bioinformatics, UK). Read trimming was performed using the software Trim Galore (version 0.4.4, Babraham Bioinformatics, UK) with the following parameters: –*paired*, *–quality* 20, –*length* 50, –*clip_R1* 10, –*clip_R2* 10, –*three_prime_clip_R1* 20, and –*three_prime_clip_R2* 20. After processing, paired-end sequencing reads were mapped to the latest bovine reference genome (ARS-UCD1.2) using the software Hisat2 (v2.1.0)^65^.

Read alignments were provided to Cufflinks (version 2.2.1) in order to construct transcript models^66^. Sample assemblies were merged together with the ARS-UCD1.2 bovine genome assembly annotation file using Cuffmerge (Cufflinks, version 2.2.1) in order to combine novel transcripts with known annotated transcripts. The number of reads that mapped to each annotated gene in the final transcriptome assembly was obtained using the python script *htseq-count* (v0.6.1p1) using the option *intersection-nonempty*^67^.

Genes with read counts $<$ 10 in at least 4 biological replicates were removed from the raw expression data and not included in the statistical analysis. Differentially expressed genes between treatments were detected using the *R* package edgeR^68^. This *R* package combines (i) the use of the trimmed mean of M-values as normalization method, (ii) an empirical Bayes approach for estimating tagwise negative binomial dispersion values, and finally (iii) generalized linear models and likelihood ratio tests for detecting differentially expressed genes. All the analyses were performed using the default settings for all parameters.

The enrichment of Gene Ontology (GO) and Kyoto Encyclopedia of Genes and Genomes (KEGG)^69^ terms with differently expressed genes was analyzed using Fisher’s exact test, a hypergeometric-based overrepresentation test commonly used to evaluate 2 × 2 contingency tables. Differentially expressed genes (*P*-value $\le$ 0.01) that had ENSEMBL annotations were tested against the background set of all expressed genes with ENSEMBL annotations. Genes were assigned to GO and KEGG terms using the function *getBM* from the *R* package biomaRt (v 2.36.1)^25^ and the function HyperGEnrich from the *R* package EnrichKit (v 1.1.383; https://github.com/liulihe954/EnrichKit), respectively. The Fisher’s exact test was implemented using the function *fisher.test* in the *R* software.

Five DEG in adipose tissue (*ANKRD33B, CLSTN3, CYP4F2, IFI47* and *REEP6*), and four DEG in muscle tissue (*CISH, CCN2, EGR1* and *FOS*) were selected for validation using quantitative real time PCR (CFX96 Touch Real-Time PCR Detection System). Selection criteria included genes with *P* < 2.0 × 10^–4^. The same RNA samples used for RNA-Seq were used for gene expression validation using quantitative real-time PCR. The expression of serotonin receptors (*5-HTR1A, -1B, -1D, -1F, -2A, -2B, -2C, -3A, -3B, -3C, -4, -5A, -6,* and *-7*) and genes involved in serotonin synthesis and metabolism (*SERT* and *TPH1*) was measured in adipose and muscle. A more detailed description of RNA extraction, cDNA synthesis, primer design, primer sequence information, and validation analysis can be found in Supplementary Methods.

Hematoxylin and eosin (H&E) staining was performed to visualize adipose and muscle tissue microstructure. The EVOS XL Core imaging system (Advanced Microscopy Group, Bothell, WA) was used to take three photomicrographs (40× magnification) for quantification of adipocyte number and area, and muscle fiber area using ImageJ software^70^ and the point picker plugin. To quantify cell proliferation, 4 µm sections were de-paraffinized, and were treated by Citra Steam (Biogenex, Fremont, CA; #HK086-9K) for 30 min. Background Sniper (Biocare Medical, Walnut Creek, CA; #BS966M) was used for 15 min to reduce unspecific background staining. Sections were incubated with Mouse anti-Human Ki67 (1:100, DAKO #M7240, clone MIB-1) 60 min, stain was visualized using Mach 2 Mouse HRP polymer (Biocare Medical, Walnut Creek, CA; # MHRP520L), and following the DAB chromagen (Vector Laboratories, Burlingame, CA; #SK-4105) and CAT hematoxylin counterstain (Biocare Medical, Walnut Creek, CA; #CATHE-M). Total tissue area for photomicrographs taken at 40× is 96,820 µm^2^. The proportion of proliferating nuclei in the adipose and muscle tissue was calculated as the total number of positive stained nuclei across all 3 fields divided by the total nuclei (positive and negative) in all 3 fields. Histological data were analyzed by analysis of variance (ANOVA) using the MIXED procedure of SAS and GLIMMIX procedure for proportion data (v. 9.4 SAS Institute Inc., Cary, NC). The model included the fixed effect of treatment (CON vs. 5-HTP), and the calf ID nested within treatment as a random effect. Data are presented as least square means ± standard error of the mean (LS means ± SEM). Statistical significance was declared at *P* ≤ 0.05 and tendencies at 0.05 < *P* ≤ 0.10.

## Supplementary Information

Supplementary Information.
