# Supplementary material for: Histological and transcriptomic analysis of adipose and muscle of dairy calves supplemented with 5-hydroxytryptophan
Source: Sci Rep. 2021 May 6;11:9665. doi: 10.1038/s41598-021-88443-w (PMC8102591; doi:10.1038/s41598-021-88443-w)
Supplement: Supplementary file 1 — Supplementary Information. [file 41598_2021_88443_MOESM1_ESM.docx]

**Supplemental Figure 1.** Gene expression of serotonin receptors (*5-HTR1A, -1B, -1D, -1F, -2A, -2B, -2C, -3A, -3B, -3C, -4, -5A, -6,* and *-7*) and genes involved in serotonin synthesis and metabolism (*SERT* and *TPH1*) in the (**A**) adipose and (**B**) muscle tissue. The geometric mean of three housekeeping genes for adipose (*B- Actin, RSP-18, UXT*) and two for muscle (*HRPT-1*, *UXT*) was used to normalize gene expression (∆Ct) to calculate gene expression as Fold Change (2^∆∆Ct^). Primer sequences can be found in Field et al. (2021)^1^ Statistical significance declared at *P* ≤ 0.05 and receptors were found not different compared to control.


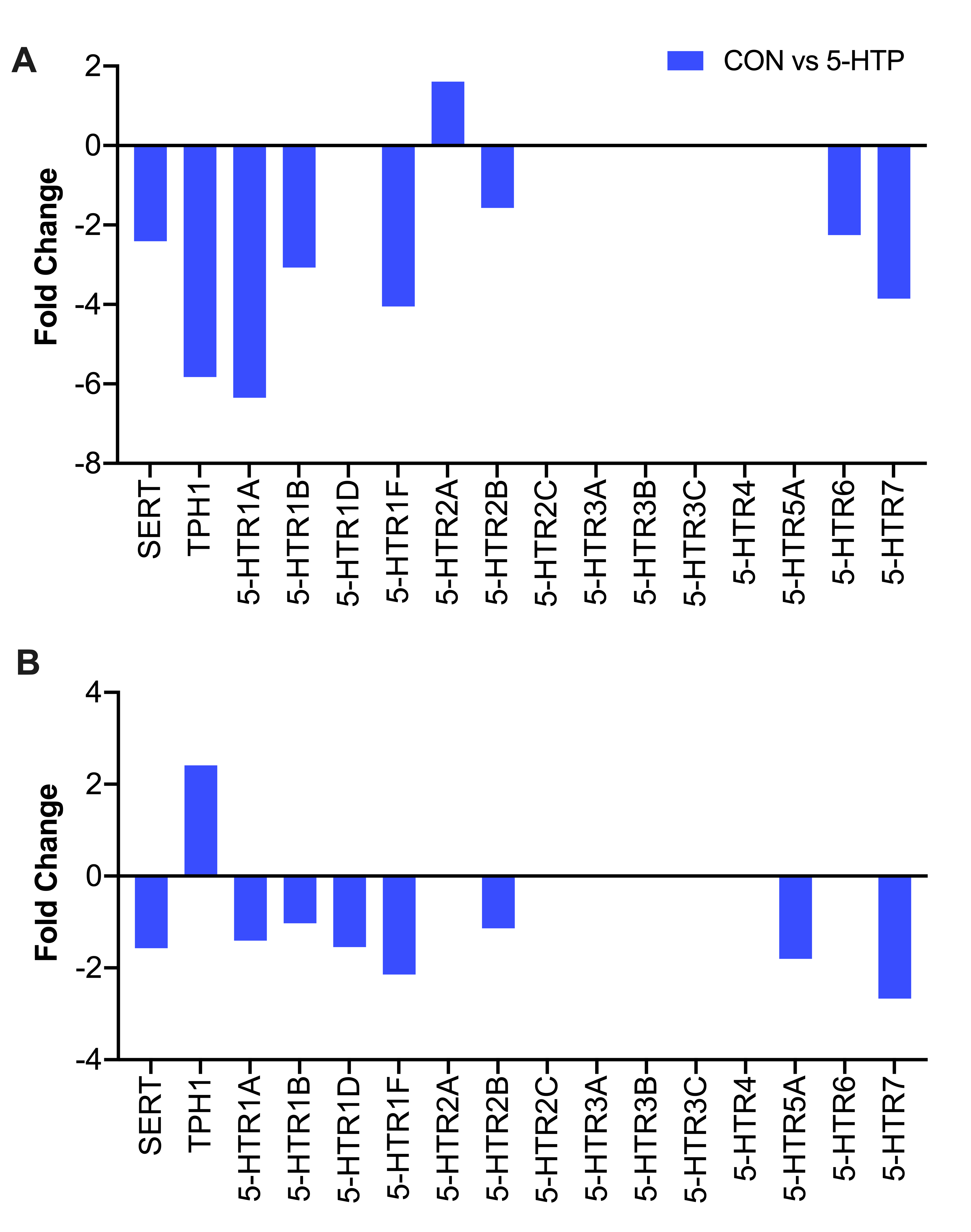


**Supplemental Figure 2.** Circulating (**A**) serotonin, (**B**) non-esterified fatty acids (NEFA), (**C**) insulin, and (**D**) glucose in blood plasma. Samples were measured in duplicate using an enzyme immunoassay kit for serotonin (IM1749; Immunotec, Beckman Coulter, Marseille Cedex 9, France), an enzymatic colorimetric assay for NEFA (Wako HR Series NEFA-HR(2), Chuo-Ku, Japan; # 99534791), a commercial ELISA (Mercodia Bovine Insulin ELISA, Uppsala, Sweden; #10120101) and a colorimetric assay (Wako Autokit Glucose, Chuo-Ku, Japan; #99103021) all according to manufacturer’s instructions. Serotonin data is adapted from Marrero et al. (2019)^24^. Insulin and glucose data is adapted from Field et al. (2021).^1^ Data was analyzed by analysis of variance using the MIXED procedures of SAS. Model included fixed effect of treatment (CON, 5-HTP), time (repeated measures) and their interaction. Calf ID nested within treatment was used as a random effect. Data presented as LS means ± SEM and statistical significance declared at *P* ≤ 0.05 denoted (*). No differences were found in circulating NEFA and glucose concentrations.


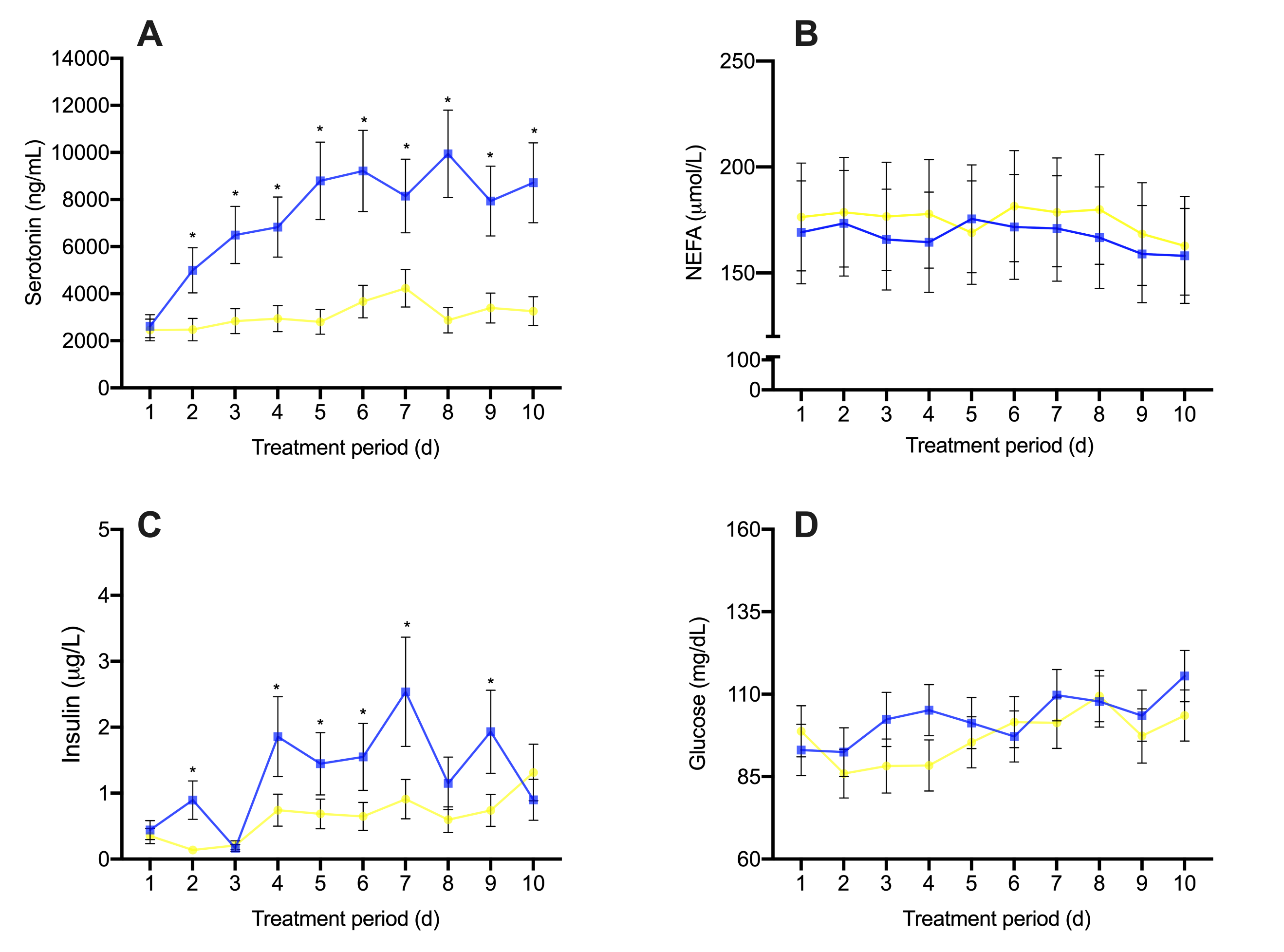


**Supplemental Figure 3.** RNA-Sequencing (RNA-Seq) validation by quantitative real-time PCR (qPCR). Comparison of the log_2_ fold change of RNA-Seq and qPCR in (**A**) adipose tissue for five differentially expressed genes (*ANKRD33B, CLSTN3, CYP4F2, IFI47* and *REEP6*) and (**B**) muscle tissue for four differentially expressed genes (*CISH, CCN2, EGR1* AND *FOS*) when comparing pre-weaned dairy calves supplemented 10 days with 5-Hydroxytrytophan (5-HTP, *n* = 4) vs. no-supplemented calves (CON, *n* = 4)*.* The y-axis displays the -log_2_ fold change of each gene and the x-axis lists the gene symbol.


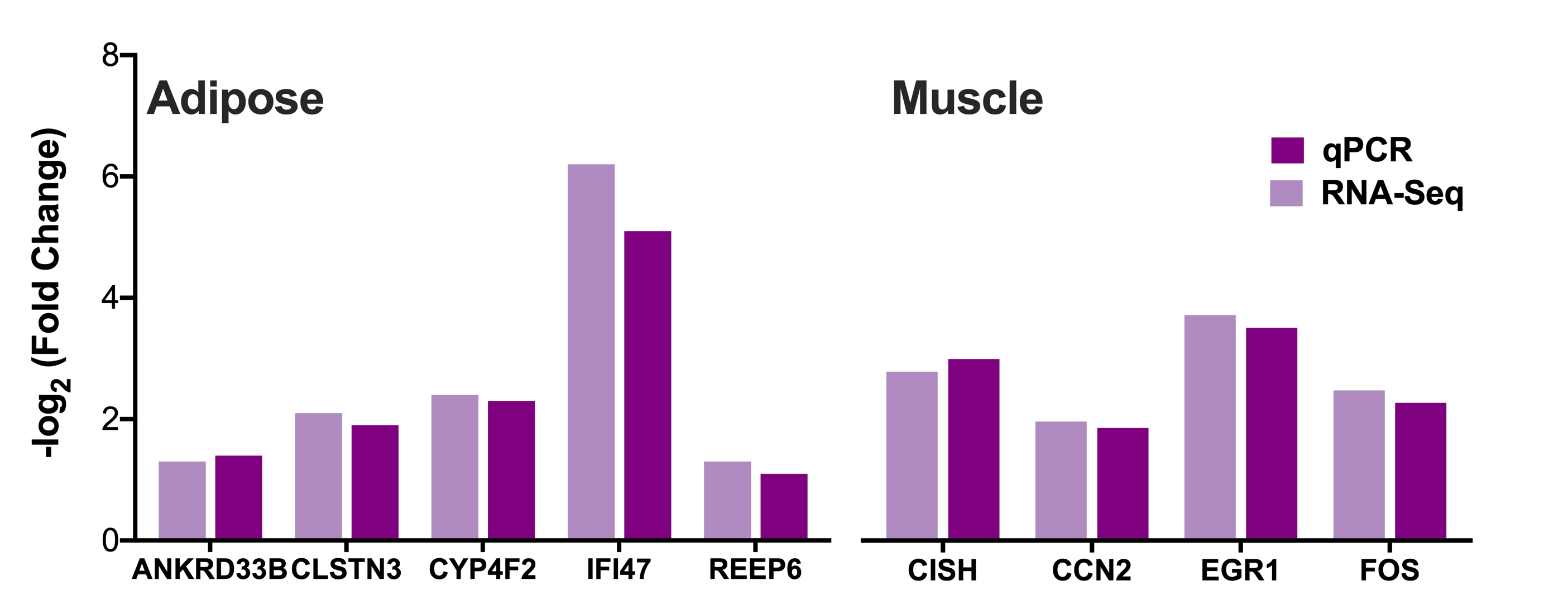


**Supplemental Figure 4.** Average daily gain (ADG) over the 10-day treatment period following 5-hydroxytryptophan (5-HTP) supplementation to pre-weaned dairy calves. ADG data is adapted from Marrero et al. (2019).^2^


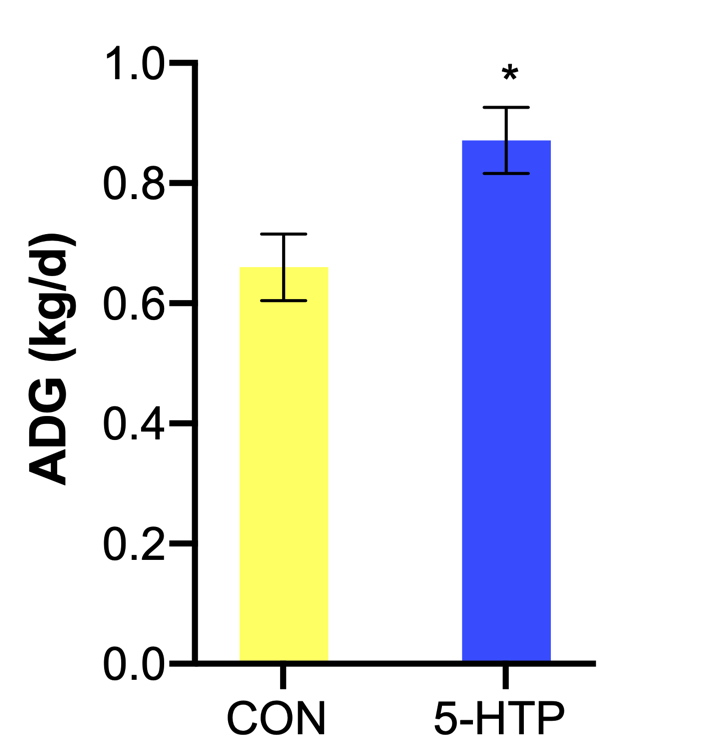


**Supplemental Table 1**. Differentially expressed genes (DEG, FDR ≤ 20%) in adipose tissue of pre-weaned dairy calves supplemented with 5-hydroxytrytophan (5-HTP, 90 mg/d; *n* = 4) or saline (CON, 8 mL saline; *n* = 4) for 10 consecutive days. A total of 22 DEGs were identified, of which 11 where upregulated and 11 where downregulated (5-HTP vs. CON). LogFC (Fold Change) and logCPM (Counts Per Million).

| **Gene** | **Gene Name** | **Full Name** | **logFC** | **logCPM** | **PValue** | **FDR** |
| --- | --- | --- | --- | --- | --- | --- |
| XLOC_040296 |  |  | -5.2 | 2.7 | 1.7E-05 | 5.2E-02 |
| XLOC_040580 |  |  | -5.0 | 2.2 | 1.8E-04 | 1.7E-01 |
| XLOC_027957 |  |  | -4.0 | -0.8 | 5.8E-06 | 2.9E-02 |
| ENSBTAG00000046516 | FUT7 | Fucosyltransferase 7 | -2.4 | 0.1 | 2.9E-05 | 6.9E-02 |
| ENSBTAG00000008129 | CLSTN3 | Calsyntenin 3 (calcium ion binding) | -2.1 | 3.4 | 9.8E-06 | 3.7E-02 |
| ENSBTAG00000050527 | Novel gene |  | -1.8 | 1.0 | 1.2E-04 | 1.3E-01 |
| XLOC_040763 |  |  | -1.8 | 2.6 | 1.5E-04 | 1.5E-01 |
| XLOC_025922 |  |  | -1.4 | 2.4 | 9.8E-05 | 1.2E-01 |
| ENSBTAG00000040193 | COLQ | Collagen tail subunit of asymmetric acetylcholinesterase | -1.2 | 5.6 | 7.3E-05 | 1.1E-01 |
| ENSBTAG00000051541 | COLQ | Collagen tail subunit of asymmetric acetylcholinesterase | -1.2 | 5.6 | 7.3E-05 | 1.1E-01 |
| ENSBTAG00000047029 | Novel gene |  | -1.2 | 4.1 | 7.9E-05 | 1.1E-01 |
| ENSBTAG00000002311 | REEP6 | Receptor accessory protein 6 | 1.3 | 4.4 | 2.0E-04 | 1.8E-01 |
| ENSBTAG00000011337 | ANKRD33B | Ankyrin repeat domain 33B | 1.3 | 6.4 | 6.7E-05 | 1.1E-01 |
| XLOC_024071 |  |  | 1.3 | 3.3 | 1.9E-05 | 5.2E-02 |
| XLOC_028032 |  |  | 1.9 | 2.6 | 1.1E-04 | 1.2E-01 |
| ENSBTAG00000014220 | Novel gene | CD36 molecule (thrombospondin receptor) | 2.4 | 0.1 | 4.4E-05 | 9.3E-02 |
| ENSBTAG00000039319 | CYP4F2 | Cytochrome P450, family 4, subfamily F, polypeptide 2 | 2.4 | 1.7 | 9.4E-05 | 1.2E-01 |
| XLOC_005436 |  |  | 2.6 | 5.2 | 6.4E-05 | 1.1E-01 |
| XLOC_028208 |  |  | 2.6 | 1.4 | 3.0E-07 | 2.8E-03 |
| XLOC_002658 | SLC7A8 |  | 3.0 | 2.7 | 6.1E-06 | 2.9E-02 |
| XLOC_025101 |  |  | 3.0 | 0.0 | 1.4E-04 | 1.4E-01 |
| ENSBTAG00000015727 | IFI47 | Interferon gamma inducible protein 47 | 6.2 | 3.4 | 5.2E-18 | 9.8E-14 |

**Supplemental Table 2.**  Differentially expressed genes (DEG, FDR ≤ 20%) in muscle tissue of pre-weaned dairy calves supplemented with 5-hydroxytrytophan (5-HTP, 90 mg/d; *n* = 4) or saline (CON, 8 mL saline; *n* = 4) for 10 consecutive days. A total of 31 DEGs were identified, of which 11 where upregulated and 21 where downregulated (5-HTP vs. CON). LogFC (Fold Change) and logCPM (Counts Per Million).

| **Gene** | **Gene Name** | **Full Name** | **logFC** | **logCPM** | **PValue** | **FDR** |
| --- | --- | --- | --- | --- | --- | --- |
| ENSBTAG00000010069 | EGR1 | Early growth response 1 | -3.6 | 7.2 | 2.3E-05 | 4.6E-02 |
| ENSBTAG00000022622 | CISH | Cytokine inducible SH2 containing protein | -2.7 | 6.5 | 2.7E-07 | 1.0E-03 |
| ENSBTAG00000004322 | FOS | Fos proto-oncogene | -2.4 | 5.1 | 2.4E-05 | 4.6E-02 |
| ENSBTAG00000003196 | PAPSS2 | 3,-phosphoadenosine 5,-phosphosulfate synthase 2 | -2.3 | -0.7 | 3.9E-05 | 6.6E-02 |
| ENSBTAG00000007665 | NPR3 | Natriuretic peptide receptor 3 | -2.0 | 3.8 | 3.1E-06 | 7.8E-03 |
| ENSBTAG00000038496 | CR2 | Complement C3d receptor 2 | -2.0 | 0.7 | 2.9E-04 | 1.6E-01 |
| ENSBTAG00000005419 | AOC1 | Amine oxidase copper containing 1 | -2.0 | 2.0 | 9.3E-05 | 8.4E-02 |
| ENSBTAG00000006367 | CCN2 | Cellular communication network factor 2 | -1.9 | 0.7 | 1.6E-04 | 1.2E-01 |
| XLOC_012144 |  |  | -1.8 | -0.5 | 7.9E-05 | 7.6E-02 |
| ENSBTAG00000011196 | C1QB | Complement C1q B chain | -1.6 | 2.8 | 2.9E-04 | 1.6E-01 |
| ENSBTAG00000013167 | SIGLEC1 | Sialic acid binding Ig like lectin | -1.5 | 0.4 | 4.0E-04 | 1.9E-01 |
| ENSBTAG00000048122 | CFD | Complement factor D | -1.4 | 1.9 | 5.8E-05 | 7.5E-02 |
| ENSBTAG00000004840 | C1S | Complement C1s | -1.3 | 5.3 | 3.9E-04 | 1.9E-01 |
| XLOC_017327 | TTN | Titin | -1.3 | 5.1 | 2.5E-07 | 1.0E-03 |
| ENSBTAG00000004305 | RGS16 | Regulator of G protein signaling 16 | -1.3 | 2.8 | 3.1E-04 | 1.6E-01 |
| ENSBTAG00000022150 | MRXA5 | Matrix remodeling associated 5 | -1.2 | 3.6 | 1.9E-04 | 1.3E-01 |
| ENSBTAG00000054051 | Novel gene |  | -1.2 | 5.4 | 1.1E-08 | 8.4E-05 |
| XLOC_029760 |  |  | -1.2 | 3.3 | 2.3E-04 | 1.5E-01 |
| ENSBTAG00000046177 | IGFN1 | Immunoglobulin like and fibronectin type III domain containing 1 | -1.1 | 11.1 | 7.1E-05 | 7.5E-02 |
| ENSBTAG00000052033 | Novel gene |  | -1.0 | 5.2 | 6.7E-05 | 7.5E-02 |
| ENSBTAG00000000283 | CSF1 | Colony stimulating factor 1 | -0.9 | 3.6 | 3.9E-04 | 1.9E-01 |
| ENSBTAG00000004815 | Novel gene |  | 0.7 | 4.1 | 2.0E-04 | 1.3E-01 |
| XLOC_012438 | ASCC2 |  | 0.8 | 5.5 | 2.9E-04 | 1.6E-01 |
| ENSBTAG00000006232 | WDR86 | WD repeat domain 86 | 1.1 | 5.0 | 7.3E-05 | 7.5E-02 |
| ENSBTAG00000018453 | ANKRD37 | Ankyrin repeat domain 37 | 1.2 | 2.1 | 2.7E-06 | 7.8E-03 |
| ENSBTAG00000049695 | LMX1B | LIM homeobox transcription factor 1 beta | 1.5 | 1.0 | 1.6E-04 | 1.2E-01 |
| XLOC_030964 | WDR86 |  | 1.6 | 4.7 | 5.7E-05 | 7.5E-02 |
| XLOC_017727 |  |  | 2.0 | 0.3 | 3.1E-04 | 1.6E-01 |
| XLOC_018883 |  |  | 2.1 | -0.5 | 1.0E-04 | 8.8E-02 |
| XLOC_039781 |  |  | 2.1 | -0.8 | 1.4E-04 | 1.1E-01 |
| XLOC_040380 |  |  | 2.1 | 0.3 | 6.6E-05 | 7.5E-02 |
| XLOC_029934 |  |  | 2.5 | -0.1 | 2.7E-04 | 1.6E-01 |
| ENSBTAG00000015727 | IFI47 | Interferon gamma inducible protein 47 | 6.8 | 1.5 | 2.5E-19 | 3.8E-15 |

**Supplemental Table 3.** Primer sequences used for validation of RNA-Seq results. All primer sequences were specifically designed for this study to span exon-exon junctions, to minimize the potential of amplifying genomic DNA, using Primer3 software with sequences obtained from GenBank (http://www.ncbi.nlm.nih.gov/). All primer pairs displayed melting curves with a single peak, indicative of a pure, single amplicon, confirmed the specificity of the primers.

| **Gene name and symbol** | **Accession number** | **Forward Primer (5' --> 3')** | **Reverse Primer (3' --> 5')** | **Tissue** |
| --- | --- | --- | --- | --- |
| Ankyrin repeat domain 33B (*ANKRD33B*) | NM_001205847.3 | CCTTATTGTCGCCTGCTACC | CCACTCCTGGGAGGACATAC | Adipose |
| Cytochrome P450 family 4 subfamily F polypeptide 2 (*CYP4F2*) | XM_010806565.3 | CACAGCCAGTGGTCTCTCCT | ACCGCAGACTCTCCTTGATG | Adipose |
| Calsyntenin 3 (*CLSTN3*) | NM_001075425.2 | ATACCCCCTTCCTCATCGAC | GCTTCTTCCCGCAGTCATAA | Adipose |
| Interferon gamma inducible protein 47 (*IFI47*) | NM_001034545.2 | GCACAACTCCAACAGAAGCA | CCAGAATGTGGCTGTGATTG | Adipose |
| Receptor accessory protein 6 (*REEP6*) | NM_001038135.2 | CTCCTCTGCTGTGCAGTCTC | CACAGTGTCGTCCTCTTTGC | Adipose |
| Cytokine inducible SH2 containing protein (*CISH*) | NM_001046586.1 | CTCCTACCTTCGGGAATCTG | TGCTGTCACGTACCAGGAAG | Muscle |
| Cellular communication network factor 2 (*CCN2*) | NM_174030.2 | GAGGAATGGGTGTGTGATGA | AGTTGGCTCGGATCATGGTT | Muscle |
| Early growth response 1 (*EGR1*) | NM_001045875.1 | CAGCAGCGGTAGCAGCAG | TGGGGTAACTCGTCTCCACT | Muscle |
| Fos proto-oncogene (FOS) | NM_182786.2 | AACCATGACAGGAGGCAGAG | ATTCCTTTCCCTTCGGATTC | Muscle |

**Supplemental Methods.** Adipose and muscle tissue samples (90 mg) were homogenized (Omni Tissue Master 125, GA, U.S.A.) and RNA was extracted using the RNeasy Mini Kit (Qiagen, Valencia, CA; #74104) following manufacturer’s instructions. Total RNA concentration and absorbance ratios were quantified using the Nanodrop™ (2000/2000c Spectrophotometers, Thermo Scientific ™, U.S.A.; #ND-2000) and subsequently stored at -80 °C. All samples had a A260/280 ratio > 1.8. One µg RNA was reverse transcribed with iScript Reverse Transcription Supermix (Bio-Rad, Hercules, CA; #1708841) and diluted (1:5) in UltraPure™ DNase/RNase-free distilled water (Thermo Fisher, U.S.A.; #10977015). Quantitative real-time PCR was conducted using the CFX96 Touch Real-Time PCR detection System (Bio-Rad) using SSoFast™ EvaGreen Supermix as described by Dado-Senn (2018).^3^ Primer sequences for validated genes can be found in Supplemental Table 3. Internal controls (*RSP-18* for adipose, and *HRPT-1* for muscle) were chosen based on their stable expression across all RNA-Seq samples. The association between normalized gene expression (ΔCt) and treatments was tested using a likelihood ratio test.^72^ The relative gene expression was calculated using the 2^ΔΔCt^ method ^4^

**Refererences**

1. Field SL, Marrero MG, Dado-Senn B, et al. Peripheral serotonin regulates glucose and insulin metabolism in Holstein dairy calves. Domestic Animal Endocrinology. 2021/01/01/ 2021;74:106519.
2. Marrero MG, Field SL, Skibiel AL, Dado-Senn B, Driver JP, Laporta J. Increasing serotonin bioavailability alters gene expression in peripheral leukocytes and lymphoid tissues of dairy calves. *Scientific Reports.* 2020/06/16 2020;10(1):9712.
3. Dado-Senn B, Skibiel AL, Fabris TF, et al. RNA-Seq reveals novel genes and pathways involved in bovine mammary involution during the dry period and under environmental heat stress. *Scientific reports.* 2018;8(1):11096-11096.
4. Livak KJ, Schmittgen TD. Analysis of relative gene expression data using real-time quantitative PCR and the 2^-DDCT^ method. *Methods.* Dec 2001;25(4):402-408.
